# Impact of Sickle Cell Hemoglobin Genotypes on Clinical Outcomes Among *Plasmodium falciparum* Malaria Patients in Luanda, Angola

**DOI:** 10.1002/hsr2.73288

**Published:** 2026-09-23

**Authors:** Cruz S. Sebastião, Eduardo Ekundi‐Valentim, Edson Cassinela, Euclides Sacomboio

**Affiliations:** ^1^ Centro Nacional de Investigação Científica (CNIC) Luanda Angola; ^2^ Centro de Investigação em Saúde de Angola (CISA) Instituto Nacional de Investigação em Saúde (INIS) Luanda Angola; ^3^ Global Health and Tropical Medicine, GHTM, LA‐REAL Instituto de Higiene e Medicina Tropical, IHMT, Universidade NOVA de Lisboa Lisboa Portugal; ^4^ Universidade Rainha Nginga a Mbande (URNM) Malanje Angola; ^5^ Instituto de Ciências de Saúde (ICISA) Universidade Agostinho Neto (UAN) Luanda Angola; ^6^ Laboratório de Investigação em Ciências de Saúde (LAICISA) Luanda Angola; ^7^ Universidade Gregório Semedo (UGS) Luanda Angola

**Keywords:** Angola, clinical outcomes, malaria, sickle cell disease

## Abstract

**Background:**

Sickle cell anemia (SCA) may influence malaria susceptibility and clinical outcomes in endemic regions. However, the interaction between hemoglobin genotypes and malaria severity remain poorly defined in endemic African settings, particulary in Angola, where SCA and malaria impose a substantial public health burden. Herein, we investigate associations between hemoglobin genotypes and clinical outcomes among malaria patients.

**Methods:**

This was a prospective cohort study conducted with 252 malaria patients in Luanda, the capital city of Angola, a sub‐Saharan African country. Association between hemoglobin genotypes and demographic, geographic, parasitological, and clinical variables were assessed in relation to malaria outcomes.

**Results:**

HbAA was predominant (75.0%), followed by HbAS (16.7%) and HbSS (8.3%). Mean age did not differ significantly across genotypes (*p* = 0.289), whereas gender distribution differed significantly (*p* = 0.008), with a high proportion of females among HbAS individuals (73.8%). No significant differences were observed across genotypes in residence, ABO blood group, or parasitemia levels (*p* > 0.05). Clinical outcomes differed significantly by hemoglobin genotype (*p* = 0.016), with lower discharge rates (57.1%) and higher mortality (19.0%) among HbSS patients. Unfavorable outcomes were more frequent among adults with HbS (OR: 1.59), males (OR: 2.03), peri‐urban residents (OR = 2.2), or blood group O (OR = 2.16), although no statistical significance was observed (*p* > 0.05). Among HbAA patients, low parasitaemia was significantly associated with favorable outcomes (OR = 0.35, *p* = 0.016).

**Conclusions:**

Our findings suggest that HbSS may be associated with increased malaria morbidity and mortality in Angola. Further studies incorporating molecular characterization of malaria parasites are needed to elucidate clinical trajectories and parasite‐host interactions.

## 1 Introduction

Malaria imposes a disproportionate burden on sub‐Saharan Africa, accounting for 95% of global cases and deaths, with Angola ranking among the most affected regions [1]. The interaction between malaria and host genetic factors, particularly hemoglobin variants, has emerged as a paradigm of evolutionary medicine [2, 3]. Sickle cell anemia (SCA), occurring from a single nucleotide substitution in the β‐globin gene, produces abnormal hemoglobin S (HbS) [4]. Homozygosity (HbSS) manifests as SCA, while heterozygous individuals (HbAS) typically remain asymptomatic while carrying one mutant allele [5]. The prevalence of the sickle cell allele in malaria‐endemic areas shows a balanced polymorphism, in which HbAS confers substantial protection against severe malaria caused by Plasmodium falciparum [6, 7]. Previous studies demonstrated that HbAS carriers exhibit reduced parasite densities and mortality [6], with approximately 90% protection against life‐threatening malaria in children [8]. Protective mechanisms involve enhanced phagocytosis of parasitized erythrocytes, diminished cytoadherence, impaired parasite replication in HbAS cells, and oxygen‐dependent growth inhibition [9, 10].

Individuals with HbSS experience chronic hemolytic anemia, functional asplenia, and immune dysregulation, which may worsen vulnerability to severe infections [11, 12]. Studies across diverse African populations have generated conflicting results regarding malaria susceptibility and severity in SCD patients, with some reporting increased severe disease risk while others suggest potential protection at lower parasite densities [13, 14]. ABO blood group may also influence outcomes, with group O associated with a lower risk of severe malaria, suggesting combined effects of hemoglobin genotype and ABO group [15].

In Angola, where malaria varies geographically, and SCA affects approximately 3% of newborns, understanding their intersection is critical for clinical management and public health strategy [16, 17]. To the best of our knowledge, we present the analysis of sickle cell hemoglobin genotype distribution among malaria patients and their associations with demographic characteristics, parasitemia levels, and clinical outcomes, in order to inform targeted interventions and optimize patient care in malaria patients with sickle cell hemoglobin genotypes.

## 2 Methodology

### 2.1 Study Design and Setting

We conducted a prospective cohort study with 252 hospitalized patients with malaria at two tertiary healthcare facilities (Josina Machel Hospital and Hospital Geral), both located in Luanda, the capital city of Angola, between January 2023 and December 2024. Exclusion criteria were pregnancy, cerebral malaria, diabetes, hypertension, cardiovascular disease, chronic kidney disease, HIV, and HBV/HCV infection. This study was conducted in accordance with the guidelines of the Declaration of Helsinki and approved by the Ethics Committee for Research Involving Human Subjects and the Scientific Council of the Institute of Health Sciences, Agostinho Neto University (Approval No. 118/GD/ICISA/UAN/2021, February 25, 2021). Institutional consent was also obtained from both participating hospitals, the Josina Machel Hospital (No. 36/DPC/HJM/2023) and Hospital Geral (No. 272/DPC/HGL/2023). Written informed consent was secured from all participants or their legal guardians prior to enrollment and sample collection.

### 2.2 Laboratory Procedure

Initial malaria diagnosis was performed by hospital clinicians based on standard clinical criteria. Confirmatory testing was carried out by the research team using rapid diagnostic tests (SD‐Bioline Malaria Ag Pf/PAN) and microscopic examination of blood smears. Parasitemia was estimated by counting parasites against 200 write blood cells and categorized as low (≤ 50 parasites/µL), moderate (51–1000 parasites/µL), high (1001–10,000 parasites/µL), and very high (> 10,000 parasites/µL), following WHO guidelines. Hemoglobin electrophoresis was conducted using the Sebia Minicap system with the Hb (E) Minicap kit, allowing separation of normal hemoglobins (A, F, A2) and identification and quantification of variant hemoglobins, including HbS, HbC, HbE, and HbD. HbS‐containing genotypes were defined as HbAS and HbSS, representing heterozygous and homozygous HbS states, respectively. Peripheral venous blood samples were collected in EDTA tubes for ABO blood typing. Blood group phenotyping was performed using a microplate agglutination assay with commercial anti‐A, anti‐B, and anti‐D sera (Immucor, Portugal) according to the manufacturer's instructions.

### 2.3 Statistical Analysis

Data were analyzed using SPSS version 31 (IBM SPSS Statistics, USA). Continuous variables with normal distribution were summarized as means with standard deviations, while categorical variables were presented as frequencies and percentages. Composite variables were created to evaluate the interaction between sickle cell hemoglobin status and demographic (age, gender, and residence), clinical (ABO blood group), and parasitological (parasitemia level) factors in relation to clinical outcomes. Clinical outcomes were classified as favorable (discharge) or unfavorable, defined as death or prolonged hospitalization during treatment. Associations between categorical variables were assessed using chi‐square or Fisher's exact tests, and univariate logistic regression was applied when appropriate. Statistical significance was defined as *p* < 0.05.

## 3 Results

### 3.1 Sickle Cell Hemoglobin Genotypes Distribution With Demographic and Clinical Features

Table 1 presents the distribution of sickle cell hemoglobin genotypes and putative association with demographic, parasitological, and clinical characteristics among malaria patients in Angola. A total of 252 individuals were included in the analysis. The HbAA genotype was the most prevalent (75.0%), followed by HbAS (16.7%) and HbSS (8.3%). The overall mean age was 30.3 ± 16.0 years. Age did not differ significantly between hemoglobin genotypes (*p *= 0.289), although individuals with the HbSS genotype (34.1 ± 19.9) had a higher mean age compared to HbAA (29.4 ± 15.7) and HbAS (32.4 ± 14.8). Most participants were adults aged ≥ 18 years (87.7%), with no significant variation between genotype groups (*p* = 0.267). The distribution by gender differed significantly according to sickle cell hemoglobin genotypes (X^2^ = 9.62, df = 2, *p* = 0.008), with moderate magnitude (Cramer's V = 0.2). The HbAS group showed a predominance of women (73.8%), while the HbAA and HbSS genotypes showed an almost equal distribution between the genders. Residence was predominantly peri‐urban (55.2%), and no significant differences were observed among residence area distribution with genotypes (*p* = 0.976). Similarly, the distribution of genotypes did not vary significantly among ABO blood groups (*p* = 0.314), although the non‐O blood group was slightly more frequent (54.0%) than the O blood group (46.0%). Furthermore, we observed that the O blood group was more common among individuals with the SS genotype (61.9%), although this pattern was not statistically significant. Parasitemia levels showed a similar distribution among hemoglobin genotypes (*p* = 0.308). Low parasitemia was the most frequent category (39.7%), followed by high (28.2%) and moderate (26.2%). Hyperparasitemia was uncommon (6.0%) and was not observed among individuals with the HbSS genotype. According to the hemoglobin genotype distribution, high parasitemia was more common among HbAA patients (31.2%), whereas moderate parasitemia was more frequent among HbAS and HbSS patients (19% each). Clinical outcomes differed significantly according to hemoglobin genotype (Fisher's exact test, *p* = 0.016), with moderate magnitude (Cramer V = 0.2). Hospital discharge rates were high in the HbAA (82.0%) and HbAS (83.3%) groups, but lower in the HbSS group (57.1%). Prolonged hospitalization was more frequent among individuals with the HbSS genotype (23.8%) compared with the HbAA (15.3%) and HbAS (11.9%) groups. Mortality was higher in the HbSS group (19.0%) compared with the HbAA (2.6%) and HbAS (4.8%) groups.

**Table 1:** Distribution of hemoglobin genotypes with demographic, parasitological, and clinical outcomes in malaria patients from Angola.

		Hemoglobin genotypes			
Independent variables	*N* (%)	AA (%)	AS (%)	SS (%)	*p*‐value
Overall	252 (100)	189 (75.0)	42 (16.7)	21 (8.3)	
Age, mean ± SD	30.3 ± 16.0	29.4 ± 15.7	32.4 ± 14.8	34.1 ± 19.9	0.289
Age group distribution					
< 18 yrs	31 (12.3)	26 (13.8)	2 (4.8)	3 (14.3)	0.267
≥ 18 yrs	221 (87.7)	163 (86.2)	40 (95.2)	18 (85.7)	
Gender					
Female	131 (52.0)	90 (47.6)	31 (73.8)	10 (47.6)	**0.008**
Male	121 (48.0)	99 (52.4)	11 (26.2)	11 (52.4)	
Residence					
Peri‐urban	139 (55.2)	105 (55.6)	23 (54.8)	11 (52.4)	0.976
Urbannized	113 (44.8)	84 (44.4)	19 (45.2)	10 (47.6)	
ABO blood group					
Non‐O	136 (54.0)	104 (55.0)	24 (57.1)	8 (38.1)	0.314
O	116 (46.0)	85 (45.0)	18 (42.9)	13 (61.9)	
Parasitemie level					
Low	100 (39.7)	71 (37.6)	19 (45.2)	10 (47.6)	0.308
Moderate	66 (26.2)	45 (23.8)	14 (33.3)	7 (33.3)	
High	71 (28.2)	59 (31.2)	8 (19.0)	4 (19.0)	
Hyper	15 (6.0)	14 (7.4)	1 (2.4)	0 (0.0)	
Clinical outcome					
Descharged	202 (80.2)	155 (82.0)	35 (83.3)	12 (57.1)	**0.016**
Prolonged hospitalization	39 (15.5)	29 (15.3)	5 (11.9)	5 (23.8)	
Dead	11 (4.4)	5 (2.6)	2 (4.8)	4 (19.0)	

*Note:* Bold numbers mean that the results of the test were statistically significant (*p* < 0.05).

### 3.2 Impact of Hemoglobin S Genotype With Demographic or Clinical Features on Clinical Outcomes

Table 2 presents the association between clinical outcomes and hemoglobin genotype (HbAA vs. HbS containing genotypes) in combination with demographic, geographic, and parasitological variables, including age, gender, residence, ABO blood group, and parasitemia. Overall, 80.2% of participants had favorable outcomes, and 19.8% had unfavorable outcomes. Stratification by age with genotype showed that HbAA individuals under 18 years old (80.8%, OR: 0.96, *p* = 0.934) and adults 18 years or older (82.2%, OR: 0.70, *p* = 0.322) had similar favorable outcomes. Among HbS carriers, adults population (25.9%, OR: 1.59, *p* = 0.192) showed a trend toward more unfavorable outcomes compared to those under 18 years old (20%, OR: 1.01, *p* = 0.993). In the combined gender with genotype, men (31.8%, OR: 2.03, *p* = 0.147) showed a higher proportion of unfavorable outcomes compared to women (22%, OR: 1.17, *p* = 0.711). For the combination of residence with genotype, peri‐urban HbS carriers (32.4%, OR: 2.2, *p* = 0.053) showed a trend toward worse outcomes compared to urban HbS carriers (17.2%, OR: 0.82, *p* = 0.709). In the combination of ABO blood group with genotype, HbS with blood group O (32.2%, OR: 2.16, *p* = 0.069) showed a higher proportion of unfavorable outcomes compared to patients in the non‐O group (18.8%, OR: 0.92, *p* = 0.868). Finally, the combination of parasitemia with genotype showed that low parasitemia among AA patients was significantly associated with favorable outcomes (90.1%, X^2^ = 6.19, df = 1, *p* = 0.014), with moderate magnitude (Cramer V = 0.16), showing to be a putative protective factor against unfavorable outcomes (OR: 0.35, 95%CI: 0.15–0.82, *p* = 0.016). Among HbS patients, high parasitemia (32.4%, OR: 2.2, *p* = 0.053) showed a trend toward worse outcomes compared to HbS patients with low parasitemia (17.2%, OR: 0.82, *p* = 0.709).

**Table 2:** Hemoglobin S genotype and impact of demographic, geographic, and parasitological factors on clinical outcomes in malaria patients in Angola.

Hemoglobin S genotype distribution	*N* (%)	Clinical outcome			Univariate analysis	
		Favorable (%)	Unfavorable (%)	*p*‐value	OR (95% CI)	*p*‐value
Overall	252 (100)	202 (80.2)	50 (19.8)			
HbS and age group						
AA with < 18 yrs	26 (10.3)	21 (80.8)	5 (19.2)	1.000	0.96 (0.34–2.68)	0.934
AA with ≥ 18 yrs	163 (64.7)	134 (82.2)	29 (17.8)	0.322	0.70 (0.37–1.32)	0.271
HbS with < 18 yrs	5 (2.0)	4 (80.0)	1 (20.0)	1.000	1.01 (0.11–9.24)	0.993
HbS with ≥ 18 yrs	58 (23.0)	43 (74.1)	15 (25.9)	0.194	1.59 (0.79–3.17)	0.192
HbS and gender						
AA with female	90 (35.7)	72 (80.0)	18 (20.0)	1.000	1.02 (0.53–1.94)	0.962
AA with male	99 (39.3)	83 (83.8)	16 (16.2)	0.261	0.68 (0.35–1.30)	0.240
HbS with female	41 (16.3)	32 (78.0)	9 (22.0)	0.674	1.17 (0.52–2.63)	0.711
HbS with male	22 (8.7)	15 (68.2)	7 (31.8)	0.161	2.03 (0.78–5.28)	0.147
HbS and residence						
AA with periurban	105 (41.7)	85 (81.0)	20 (19.0)	0.873	0.92 (0.49–1.73)	0.790
AA with urban	84 (33.3)	70 (83.3)	14 (16.7)	0.407	0.73 (0.37–1.45)	0.373
HbS with periurban	34 (13.5)	23 (67.6)	11 (32.4)	0.063	2.20 (0.99–4.87)	0.053
HbS with urban	29 (11.5)	24 (82.8)	5 (17.2)	0.809	0.82 (0.30–2.28)	0.709
HbS with ABO blood group						
AA with non‐O	104 (41.3)	83 (79.8)	21 (20.2)	1.000	1.04 (0.55–1.95)	0.907
AA with O	85 (33.7)	72 (84.7)	13 (15.3)	0.243	0.63 (0.32–1.27)	0.199
HbS with non‐O	32 (12.7)	26 (81.3)	6 (18.8)	1.000	0.92 (0.36–2.38)	0.868
HbS with O	31 (12.3)	21 (67.7)	10 (32.3)	0.089	2.16 (0.94–4.93)	0.069
HbS and parasitemia						
AA with low parasitemia	71 (28.2)	64 (90.1)	7 (9.9)	**0.014**	0.35 (0.15–0.82)	**0.016**
AA with high parasitemia	118 (46.8)	91 (77.1)	27 (22.9)	0.272	1.43 (0.77–2.67)	0.257
HbS with low parasitemia	29 (11.5)	24 (82.8)	5 (17.2)	0.809	0.82 (0.30–2.28)	0.709
HbS with high parasitemia	34 (13.5)	23 (67.6)	11 (32.4)	0.063	2.20 (0.99–4.87)	0.053

*Note:* Bold numbers mean that the results of the test were statistically significant (*p* < 0.05). Clinical outcomes were classified as favorable (discharge) or unfavorable (death or prolonged hospitalization during treatment).

## 4 Discussion

Our study reveals disparities in clinical malaria outcomes among sickle cell hemoglobin genotypes in Angola, demonstrating that while HbAS maintains protective effects, HbSS substantially increases mortality and morbidity, with profound implications for clinical management and public health policies in malaria‐endemic African regions.

The observed distribution of hemoglobin genotypes (HbAA: 75.0%, HbAS: 16.7%, HbSS: 8.3%) aligns with patterns observed in malaria‐endemic African populations, where the prevalence of sickle cell trait generally ranges from 10% to 40%, and homozygous SCA affects 1%–3% of newborns [18]. This distribution reflects the evolutionary maintenance of the sickle cell allele through heterozygote advantage, despite the high adaptive cost of homozygous disease [2, 6].

The female predominance in the HbAS group (73.8%) warrants further investigation, possibly reflecting sampling variations, distinct survival patterns, or gender‐specific factors influencing healthcare‐seeking behavior [19]. The mean age was higher among HbSS individuals (34.1 years) compared to HbAA (29.4 years) and HbAS (32.4 years) patients, although without any statistically significant findings (*p* = 0.289), this may reflect differences in survival or healthcare access across hemoglobin genotypes. In fact, previous studies have shown that adults with sickle cell anemia who survive into their fourth decade of life may represent a select cohort with milder disease phenotypes, beneficial genetic modifiers such as elevated fetal hemoglobin levels, or better access to healthcare [20]. However, this observation contrasts with the historically high infant mortality in African populations with sickle cell anemia, suggesting a different survival pattern in Angola.

Our findings show a sevenfold increase in mortality among patients with HbSS (19.0%) compared to HbAA (2.6%) and HbAS (4.8%) individuals, highlighting the extreme vulnerability of sickle cell disease patients to severe malaria complications in this group [11, 12]. Multiple pathophysiological mechanisms underlie this increased mortality, as chronic hemolytic anemia is exacerbated by malaria‐induced hemolysis, potentially precipitating severe anemia and cardiovascular decompensation [21]. Furthermore, studies show that functional asplenia, common in most sickle cell disease patients, impairs immune responses to bloodborne pathogens, including *Plasmodium* species [22]. Indeed, the substantial increase in the rate of prolonged hospitalization among HbSS individuals (23.8%) versus 15.3% of HbAA and 11.9% of HbAS reflects these complex and overlapping pathologies requiring prolonged clinical management. Our findings of prolonged hospitalization in groups of patients with HbSS corroborate reports from Nigeria and Tanzania that document high rates of severe illness and mortality in patients with SCA and malaria [11, 23].

In contrast to the results observed in HbSS individuals, those with HbAS show favorable results comparable to those of HbAA individuals (discharge rates of 83.3% *vs.* 82.0%), corroborating the well‐established protective effect of the sickle cell trait against severe malaria [7, 8]. However, the slightly elevated mortality in HbAS patients (4.8%) compared to HbAA patients (2.6%), although not statistically significant, suggests that the sickle cell trait may not confer absolute protection in all clinical contexts, which is consistent with recent evidence indicating variable protection depending on age, transmission intensity, and disease severity [24].

The significant association between low parasitemia in HbAA patients and favorable outcomes (OR: 0.35, *p* = 0.016) confirms the established relationship between parasite load and disease severity [25]. Notably, hyperparasitemia was absent among HbSS individuals, potentially reflecting rapid progression to severe disease at lower parasite densities due to compromised host defenses, early seeking of medical attention motivated by symptom severity, or enhanced parasite clearance in the context of chronic hemolysis and splenic dysfunction [11]. The observation that moderate parasitemia predominated in sickle cell trait carriers (whether HbAS or HbSS) compared to high parasitemia in HbAA patients is aligned with experimental evidence demonstrating impaired parasite growth in HbS‐containing erythrocytes [9]. However, lower parasitemia in HbSS patients did not translate into better outcomes, indicating that factors beyond parasite density, such as baseline hemoglobin levels, immune competence, inflammatory responses, and pre‐existing organ damage, might determine clinical outcomes in patients with sickle cell disease and malaria [12].

The trend toward worse outcomes among HbS carriers with blood type O (OR: 2.16, *p* = 0.069) observed in our study deserves attention, since blood type O typically confers protection against severe malaria by reducing rosette formation and cytoadherence [25]. Therefore, this negative epistatic interaction between HbS and blood type O in determining clinical outcomes of malaria represents a poorly studied phenomenon that deserves further investigation in larger cohorts of patients with different degrees of infection and severity of malaria.

Similarly, the trend toward worse outcomes among HbS carriers in peri‐urban areas (OR: 2.2, *p* = 0.053) compared to urban residents may reflect differentiated access to health services, socioeconomic disparities, or variations in the intensity of malaria transmission and acquired immunity [16]. It is worth mentioning that urban populations can benefit from proximity to specialized SCA centers and faster access to antimalarial treatment, which can mitigate unfavorable clinical outcomes. Therefore, geographic, socioeconomic, and environmental determinants should be considered when developing malaria management strategies in patients with SCA.

Our study has some limitations. Firstly, the small sample size, particularly for the SS group, reduces statistical power and could explain the little statistical significance in most of the variables studied. Secondly, we did not assess genetic modifiers of SCA severity, such as fetal hemoglobin levels, which could influence malaria outcomes. Finally, parasite density estimates may have been influenced by variations in white blood cell counts, particularly among patients with SCA. Despite these weaknesses, the combined analysis of sickle cell hemoglobin genotypes with demographic, geographic, parasitological, and clinical variables in an endemic malaria setting carried out in this study allowed the identification of specific patterns of sickle cell hemoglobin genotypes and clinically relevant interactions rarely explored in Angolan populations. However, further studies with follow‐up in a larger sample, with molecular characterization of parasite species and description of drug resistance markers, should be carried out in populations from different regions in Angola.

## 5 Conclusion

Our findings demonstrated that HbSS could increase malaria mortality and morbidity in Angola, with a seven‐fold higher mortality rate and increased prolonged hospitalization. Moreover, patients with SCA were a vulnerable population requiring urgent attention and targeted clinical management strategies. However, larger prospective studies across Angola, incorporating parasite molecular characterization and drug‐resistance markers, are needed to better understand clinical outcomes and parasite‐host interactions.

## Author Contributions

**Cruz S. Sebastião:** conceptualization, data curation, formal analysis, methodology, investigation, writing – original draft, writing – review and editing. **Eduardo Ekundi‐Valentim:** investigation. **Edson Cassinela:** investigation. **Euclides Sacomboio:** conceptualization, data curation, formal analysis, funding acquisition, methodology, investigation, project administration, supervision, validation, writing – original draft, writing – review and editing.

## Ethics Statement

This study was conducted in accordance with the guidelines of the Declaration of Helsinki and approved by the Ethics Committee for Research Involving Human Subjects and the Scientific Council of the Institute of Health Sciences at Agostinho Neto University (Approval No. 118/GD/ICISA/UAN/2021), Hospital Josina Machel (No. 36/DPC/HJM/2023) and Hospital Geral (No. 272/DPC/HGL/2023).

## Consent

The authors have nothing to report.

## Conflicts of Interest

The authors declare no conflicts of interest.

## Transparency Statement

The lead/Corresponding author (ES) affirms that this manuscript is an honest, accurate, and transparent account of the study being reported; that no important aspects of the study have been omitted; and that any discrepancies from the study as planned (and, if relevant, registered) have been explained.

## Data Availability

The data that support the findings of this study are available on request from the corresponding author. The data are not publicly available due to privacy or ethical restrictions. The datasets used and/or analyzed during the current study are available from the corresponding author on reasonable request.
